# Herpes Simplex Viral Infection Doubles the Risk of Dementia in a Contemporary Cohort of Older Adults: A Prospective Study

**DOI:** 10.3233/JAD-230718

**Published:** 2024-02-13

**Authors:** Erika Vestin, Gustaf Boström, Jan Olsson, Fredrik Elgh, Lars Lind, Lena Kilander, Hugo Lövheim, Bodil Weidung

**Affiliations:** a Department of Public Health and Caring Sciences, Clinical Geriatrics, Uppsala University, Uppsala, Sweden; b Centre for Clinical Research, Västmanland and County Hospital, Uppsala University, Västerås, Sweden; c Department of Clinical Microbiology, Umeå University, Umeå, Sweden; d Department of Medical Sciences, Acute and Internal Medicine, Uppsala University, Uppsala, Sweden; e Department of Community Medicine and Rehabilitation, Geriatric Medicine, Umeå University, Umeå, Sweden

**Keywords:** Aged 80 and over, Alzheimer disease, apolipoprotein E, cognitive disorder, cohort study, cytomegalovirus, dementia, Herpes simplex, human herpesvirus 1, neurocognitive disorder

## Abstract

**Background:**

Evidence indicates that herpes simplex virus (HSV) participates in the pathogenesis of Alzheimer’s disease (AD).

**Objective:**

We investigated AD and dementia risks according to the presence of herpesvirus antibodies in relation to anti-herpesvirus treatment and potential *APOE* ɛ4 carriership interaction.

**Methods:**

This study was conducted with 1002 dementia-free 70-year-olds living in Sweden in 2001–2005 who were followed for 15 years. Serum samples were analyzed to detect anti-HSV and anti-HSV-1 immunoglobulin (Ig) G, anti-cytomegalovirus (CMV) IgG, anti-HSV IgM, and anti-HSV and anti-CMV IgG levels. Diagnoses and drug prescriptions were collected from medical records. Cox proportional-hazards regression models were applied.

**Results:**

Cumulative AD and all-cause dementia incidences were 4% and 7%, respectively. Eighty-two percent of participants were anti-HSV IgG carriers, of whom 6% received anti-herpesvirus treatment. Anti-HSV IgG was associated with a more than doubled dementia risk (fully adjusted hazard ratio = 2.26, *p* = 0.031). No significant association was found with AD, but the hazard ratio was of the same magnitude as for dementia. Anti-HSV IgM and anti-CMV IgG prevalence, anti-herpesvirus treatment, and anti-HSV and -CMV IgG levels were not associated with AD or dementia, nor were interactions between anti-HSV IgG and *APOE* ɛ4 or anti-CMV IgG. Similar results were obtained for HSV-1.

**Conclusions:**

HSV (but not CMV) infection may be indicative of doubled dementia risk. The low AD incidence in this cohort may have impaired the statistical power to detect associations with AD.

## INTRODUCTION

According to the World Health Organization, more than 55 million people had dementia in 2023 and nearly 10 million people are estimated to receive new dementia diagnoses each year [1]. The number of dementia cases is estimated to increase to 78 million in 2030 [2]. Alzheimer’s disease (AD) is the most common form of dementia globally, causing up to 70% of dementia cases [1]. Although the cause of AD is not yet known, accumulating evidence from large population-based prospective studies indicates a role of herpes simplex virus (HSV) infection in AD or dementia development [3–6].

The two viral species HSV type 1 (HSV-1) and 2 (collectively denoted HSV) are associated with orofacial and genital infection, respectively, although the distinction is not strict [7]. When studied separately, HSV-1 has been associated with dementia risk. HSV infection (HSV carriership) can be demonstrated by the presence of immunoglobulin (Ig) G in serum, and IgM detection and higher IgG levels reflect frequent reactivation [8]. On average, 80% of Swedish adults are carriers of HSV-1 (mean age 63±14 years) [9]. The AD or dementia risk has been associated with IgG prevalence [3, 6, 10], IgG levels [11–15], and IgM prevalence [4, 5, 16, 17]. In addition, interaction effects have been found between HSV and *apolipoprotein* ɛ4 (*APOE* ɛ4) [18–20], the strongest risk factor for AD [21], suggesting that the risk may be conferred by HSV in combination with *APOE* ɛ4 and possibly also other genetic factors [22–24]. HSV infection has also been associated with declines in cognitive performance [25–28].

Conversely, other studies have found no association between the IgG or IgM prevalence or IgG level and dementia or AD [4, 5, 19, 28–31], and no interaction effect between HSV and *APOE* ɛ4 in these associations [4]. One meta-analysis [32] found no support for an association but may have been unequipped to address differences in study design and the importance of age and differences therein, important factors associated with the AD risk, among cohorts. In addition, the potentially strong interaction effect with *APOE* ɛ4 was not examined in the meta-analysis [32].

Anti-herpesvirus treatment may mitigate the HSV-related AD risk [33]. It reduced this risk by 70% in anti–HSV-1 IgG–positive persons [34] and reduced the AD or dementia risk among people with symptomatic HSV infection [35–38]. Two studies found no association and yielded heterogenous results, respectively, regarding the ability of anti-herpesvirus treatment to decrease the AD or dementia risk in people diagnosed with HSV infection [39–40]. Lastly, one study showed that this treatment tended to decrease the dementia risk, although not significantly, in persons with self-reported reactivation of HSV infection [16].

Cytomegalovirus (CMV) is another herpesvirus whose association with the AD or dementia risk has been studied. A meta-analysis showed an association between elevated IgG levels, but not the IgG prevalence, and the dementia risk, although the evidence was considered to be insufficient [32]. In eight other studies, the anti-CMV IgG prevalence and level were not associated with the AD or dementia risk [4, 6, 11–12, 17, 41–43]. In addition, one of these studies showed that anti-CMV IgG interacted with the anti–HSV-1 IgG prevalence in association with increased AD risk [41], indicating a synergistic effect.

Thus, the relationship between HSV and dementia has not been elucidated fully and results obtained to date are far from unanimous. To better understand the potential effects of HSV on incident AD or dementia, suspected interactions with common co-infections, their treatment, and risk-related genes need to be considered in analyses, which has not commonly been reported together. Further, age is the strongest risk factor for dementia, which is difficult to adequately adjust for. This information may inform future intervention or prevention trials aiming to assess the effect of anti-herpesvirus treatment on the AD and dementia risk. The aim of this study was to investigate the roles of HSV, HSV-1 specifically, and CMV in AD and dementia risk, including examination of potential interactions with *APOE* ɛ4 carriership and the effects of anti-herpesvirus treatment, in a prospective cohort of same-age individuals.

## METHODS

### Study population

Participants in this study were selected from the Prospective Investigation of Vasculature in Uppsala Seniors (PIVUS) cohort. The PIVUS was initiated in 2001 at Uppsala University with the main purpose of examining the convenience of using measures of endothelial function and arterial compliance to predict future vascular events. Invitation letters were sent to 2,025 randomly selected persons living in Uppsala within 2 months of their 70^th^ birthdays, and 1,016 of these persons participated in the study (50.2% participation rate) [44]. The participants underwent assessments at the ages of 70, 75, and 80 years. At the age of 85 years, they were followed through medical records. All PIVUS participants who underwent serum sampling at baseline and for whom information on the level of education was available were included in the present study. The Regional Ethical Review Board of Uppsala (#2017/349) and the Swedish Ethical Review Authority (#2020-00241, #2020-06029) approved this study. All participants provided written informed consent.

### Measures

Participants reported their educational levels at baseline, and these levels were dichotomized as ≤9 and ≥10 years. Blood samples were collected from the participants through venipuncture. To detect anti–HSV-1, anti-HSV, and anti-CMV IgG and anti-HSV IgM, serum samples were analyzed using an ELISA method developed by Juto and Settergren [45] and modified by Lövheim et al. [5]. In addition, anti-HSV IgG levels were determined semi-quantitatively by calculating individual sample absorbance as a percentage of the absorbance at 405 nm of the positive control minus the negative control. The *APOE* ɛ4 status of subjects with available DNA was determined through genotyping with the Illumina HumanOmniExpress chip, as described previously [26, 46]. For the remaining participants for whom serum samples were available, an ELISA was used to detect the apolipoprotein E4 (apoE4) phenotype in serum. *APOE* ɛ4 carriership was thus defined by the detection of at least one *APOE* ɛ4 allele by genotyping or apoE4 in serum by phenotyping. *APOE* ɛ4 status was genotyped for 917 participants and apoE4 was phenotyped for 96 participants. An additional 23 serum samples from the cohort were phenotyped, yielding 100% agreement on apoE4 carriership with the genotype data.

An experienced specialist in medicine collected information on dementia and AD diagnoses and indications of cognitive impairment from participants’ medical records until 15 years after inclusion. An experienced geriatrician at the Memory Clinic of Uppsala University Hospital reviewed the diagnoses and classified cases as established or probable dementia or AD (not including mild cognitive impairment) according to standard clinical procedures, including neuroradiological findings and cognitive testing and the National Institute of Neurological and Communicative Diseases and Stroke and AD and Related Disorders Association criteria [47]. When differentiating between AD and other dementia disorders was not possible due to insufficient data in the medical records, cases were registered as ‘all-cause dementia’. Dates of death were collected from the Swedish population register.

A physician collected data on first-time anti-herpesvirus treatment (Anatomical Therapeutic Chemical classes J05AB01–J05AB20, J05AP01, and J05AD01) prescriptions from contemporary and archived primary care records from 1990–1994 to 5 years before end of follow-up, complemented from 2005 with nationwide prescription data from the Swedish Prescribed Drug Register, as described in detail previously [26]. To investigate whether a significant portion of prescriptions was undetected due to the non-collection of specialty medicine prescriptions before 2005, the complete medical (including specialty medicine) records of 81 participants were reviewed. As no additional prescription was found, this review was discontinued. Anti-herpesvirus treatment receipt was defined as the prescription of at least one anti-herpesvirus drug before the outcome or censoring.

### Statistical analyses

Subsamples of anti-HSV, anti–HSV-1, and anti-CMV IgG–seropositive subjects were formed, to study the effects of anti-herpesvirus treatment, anti-HSV IgM, anti-HSV levels, and anti-CMV levels, respectively. The prevalences of educational level ≤9 years, sex, *APOE* ɛ4 carriership, antiviral treatment, AD or dementia, and anti-CMV IgG positivity were compared between participants included and not included in the subsamples using the χ^2^ test. The time to event was defined as the number of days from inclusion to the diagnosis of dementia (if before the 15-year follow-up) or censoring. Censoring took place on the date of death or 15-year follow-up, whichever came first.

Kaplan–Meier survival plots were used to visualize survival according to anti-HSV IgG carriership. Cox proportional-hazards regression models were used to assess the risks of incident dementia and AD, respectively, with anti-HSV or anti-CMV IgG as primary predictors in the full sample. In the HSV and HSV1 subsamples, primary predictors were anti-herpesvirus treatment, anti-HSV IgM, and anti-HSV levels. Anti-CMV IgG levels was primary predictor in the CMV subsample. First, basic models including sex and a primary predictor were built. Next, the models were fully adjusted for education and *APOE* ɛ4 carriership. Finally, interaction terms were added to the fully adjusted models (primary predictor×*APOE* ɛ4 or anti-CMV IgG×anti-HSV IgG), when possible within the covariate limit. This limit was determined by dividing the number of participants with or without the outcome, whichever was smallest, by 10 [48]. The assumption of proportional hazards was assessed using the Shoenfeld residual test [49]. Non-linearity in models with serum antibody level covariates was assessed by plotting Martingale residuals against antibody levels. All models including anti-HSV IgG were repeated with anti–HSV-1 IgG in place of this variable. As a sensitivity analysis, the full models were repeated with the inclusion of a composite variable of incident stroke, myocardial infarction, or diabetes within the first 5 years of follow-up.

All analyses were two tailed, and *p* values ≤0.05 were regarded as significant. Complete case analysis was used. All statistical analyses were pre-planned and performed using IBM SPSS Statistics (version 27.0; IBM Corporation, Armonk, NY, USA). The matplotlib library (version 3.7.0) in Python (version 3.10.9) was used in the Spyder integrated development environment (version 5.4.1) in Anaconda Navigator (version 2.4.2 for Windows; 2016 Anaconda Inc., Austin, TX, USA) for data visualization.

## RESULTS

Fourteen of the 1016 PIVUS participants were excluded due to missing serum samples (*n* = 3) and educational level data (*n* = 11), leaving 1,002 participants in the present study. Table 1 shows the baseline characteristics of the full sample and HSV subsample according to outcome. The median follow-up time was 15.0 years (interquartile range: 13.5–15.0 years). Seventy-one (7%) participants developed all-cause dementia and 36 (4%) developed AD. In the full sample, the prevalence of anti-HSV IgG positivity was somewhat greater among persons with AD and dementia than among those without dementia (89%, 89%, and 82%, respectively). Six percent of participants in the HSV subsample had received anti-herpesvirus treatment up to the last 5 years before the end of follow-up. The proportions of participants with AD and dementia who had received anti-herpesvirus treatment at least 5 years before diagnosis were larger than that of participants without dementia who received anti-herpesvirus treatment at least 5 years before the end of follow-up (*n* = 3, 9%; *n* = 7, 11%; and *n* = 44, 6%, respectively). Differences between participants included and not included in subsamples (i.e., anti–HSV-1 seropositive versus seronegative and anti-CMV IgG–seropositive versus seronegative participants) are presented in the Supplementary Material.

**Table 1:** Baseline characteristics of the full sample and subsamples

	Total	AD	Dementia	Dementia-free
	(*n* = 1,002)	(*n* = 36)	(*n* = 71)	(*n* = 931)
ALL
Age (y), mean±SD	70.2±0.2	70.2±0.2	70.2±0.2	70.2±0.2
Follow-up time (y), median, IQR	15.0 (13.5–15.0)	12.4 (11.1–14.1)	12.4 (11.5–14.0)	15.0 (14.8–15.0)
Sex (women), *n* (%)	501 (50.0)	21 (58.3)	35 (49.3)	466 (50.1)
Education≤9 (y), *n* (%)	570 (56.9)	22 (61.1)	44 (62.0)	526 (56.5)
*APOE* ɛ4 pos, *n* (%)	299 (29.8)	17 (47.2)	29 (40.8)	270 (29.0)
Anti-HSV IgG pos, *n* (%)	825 (82.3)	32 (88.9)	63 (88.7)	762 (81.8)
Anti-HSV IgG level (AU), mean±SD	78.5±44.1	85.8±39.5	84.7±40.4	78.1±44.3
Anti-HSV1 IgG pos, *n* (%)	745 (74.4)	30 (83.3)	58 (81.7)	687 (73.8)
Anti-CMV IgG pos, *n* (%)	783 (78.1)	29 (80.6)	58 (81.7)	725 (77.9)
	Total	AD	Dementia	Dementia-free
	(*n* = 825)	(*n* = 32)	(*n* = 63)	(*n* = 762)
Subsample anti-HSV IgG pos
Age (y), mean±SD	70.2±0.2	70.2±0.2	70.2±0.2	70.2±0.2
Follow-up time (y), median, IQR	15 (12.2–15.0)	12.4 (10.9–14.1)	12.4 (11.2–14.1)	15.0 (12.7–15.0)
Sex (women), *n* (%)	418 (50.7)	19 (59.4)	32 (50.8)	386 (50.7)
Education≤9 (y), *n* (%)	483 (58.5)	21 (65.6)	41 (65.1)	442 (58.0)
*APOE* ɛ4 pos, *n* (%)	245 (29.7)	15 (46.9)	27 (42.9)	218 (28.6)
Anti-herpesvirus treatment^1^, *n* (%)	51 (6.2)	3 (9.4)	7 (11.1)	44 (5.8)
Anti-HSV IgG level (AU), mean±SD	95.4±27.4	96.6±26.3	95.4±28.2	95.4±27.4
Anti-HSV IgM pos, *n* (%)	81 (9.8)	3 (9.4)	5 (7.9)	76 (10.0)
Anti-HSV IgM level (AU), mean±SD	0.03±0.09	0.03±0.12	0.03±0.10	0.03±0.09

AD, Alzheimer’s disease; SD, standard deviation; IQR, interquartile range; *APOE* ɛ4, apolipoprotein E4; pos, positive; HSV, herpes simplex virus; IgG, immunoglobulin G; AU, arbitrary units; CMV, cytomegalovirus. ^1^Prescriptions made up to 5 years before the end of follow-up.

**Fig. 1:**
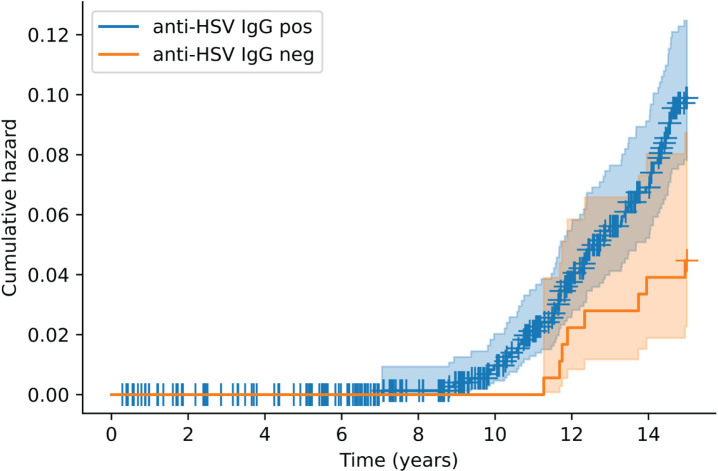
Kaplan–Meier cumulative hazard of incident dementia according to anti-HSV IgG positivity. Each + denotes a censored observation. HSV, herpes simplex virus; IgG, immunoglobulin G.

Figure 1 shows the time to dementia diagnosis according to anti-HSV IgG carriership. Anti-HSV IgG positivity doubled the risk of dementia in the basic [hazard ratio (HR) = 2.25, *p* = 0.031] and fully adjusted (HR = 2.26, *p* = 0.031) models (Table 2). No significant association between anti-HSV IgG positivity and AD was found in the full sample (Table 2). The interaction between anti-HSV IgG and *APOE* ɛ4 positivity was not significant (Table 2). Similar results were obtained for anti–HSV-1 IgG positivity (Supplementary Material).

**Table 2:** HRs for AD and dementia with anti-HSV IgG positivity and its interaction with *APOE* ɛ4 positivity for the full sample

	AD		Dementia					
	Basic model		Basic model		Fully adjusted model		Interaction model	
	HR (95% CI)	*p*	HR (95% CI)	*p*	HR (95% CI)	*p*	HR (95% CI)	*p*
Anti-HSV IgG pos	2.24 (0.79–6.33)	0.129	2.25 (1.08–4.71)	0.031	2.26 (1.08–4.72)	0.031	1.63 (0.69–3.88)	0.266
Sex (women)	1.30 (0.67–2.53)	0.432	0.90 (0.57–1.44)	0.669	0.89 (0.56–1.41)	0.614	0.88 (0.55–1.40)	0.587
Education≥10 y					0.83 (0.52–1.35)	0.460	0.84 (0.52–1.36)	0.485
*APOE* ɛ4 pos					1.73 (1.08–2.77)	0.024	0.75 (0.15–3.70)	0.720
Anti-HSV IgG pos×*APOE* ɛ4 pos							2.55 (0.48–13.66)	0.274

Data were obtained with Cox proportional-hazards regression models. Abbreviations: HR, hazard ratio; AD, Alzheimer’s disease; CI, confidence interval; HSV, herpes simplex virus; IgG, immunoglobulin G; pos, positive; *APOE* ɛ4, apolipoprotein E4.

In the HSV and HSV-1 subsamples, higher anti-HSV IgG levels were not associated with the risk of AD (HR = 1.00, 95% CI 0.99–1.02, *p* = 0.746 and HR = 1.00, 95% CI 0.99–1.02, *p* = 0.706, respectively) or dementia (fully adjusted model: HR = 1.00, 95% CI 0.99–1.01, *p* = 0.972 and HR = 1.00, 95% CI 0.99–1.02, *p* = 0.751, respectively). Anti-HSV IgM positivity was not associated with the risk of developing AD (HR = 1.00, 95% CI 0.30–3.29, *p* = 0.997 and HR = 0.62, 95% CI 0.15–2.59, *p* = 0.508, respectively) or dementia (fully adjusted model: HR = 0.95, 95% CI 0.38–2.38, *p* = 0.912 and HR = 0.72, 95% CI 0.26–2.00, *p* = 0.530, respectively). Moreover, anti-herpesvirus drug treatment was not associated with a lower risk of AD (HR = 1.46, 95% CI 0.44–4.82, *p* = 0.532 and HR = 1.64, 95% CI 0.50–5.41, *p* = 0.419, respectively) or dementia (fully adjusted model: HR = 1.70, 95% CI 0.73–3.97, *p* = 0.222 and HR = 1.60, 95% CI 0.63–4.04, *p* = 0.320, respectively) development.

In the full sample, anti-CMV IgG positivity was not associated with the AD (HR = 1.17, 95% CI 0.51–2.67, *p* = 0.717) or dementia (fully adjusted model: HR = 1.18, 95% CI 0.65–2.17, *p* = 0.586) risk. The interaction between anti-CMV and anti-HSV IgG positivity was not significant (HR = 1.63, 95% CI 0.34–7.94, *p* = 0.545). Among CMV carriers, the anti-CMV IgG level was not associated with the AD (HR = 0.99, 95% CI 0.98–1.00, *p* = 0.154) or dementia (fully adjusted model: HR = 1.00, 95% CI 0.99–1.01, *p* = 0.583) risk.

Repeating the full models with the inclusion of a composite variable of incident stroke, myocardial infarction, or diabetes produced similar results. Pearson correlation between rank of follow-up time and partial residuals of all covariates, separately, was *p* ≥ .05 for all models, indicating proportionality of hazards (Supplementary Material). Additionally, graphical assessment of covariates plotting log(-log(survival)) against log(time) for all the categorical predictors and Shoenfeld’s residuals against log(time) for continuous covariates indicated proportionality of hazards (Supplementary Material).

## DISCUSSION

In this prospective epidemiological cohort study, the seroprevalence of anti-HSV IgG was associated with a more than doubled risk of incident dementia. No significant association was found with AD, although the HR was of the same magnitude as that for dementia. No interaction between the anti-HSV IgG seroprevalence and *APOE* ɛ4 carriage or anti-CMV IgG seroprevalence with respect to the dementia risk was found. Furthermore, the anti-HSV IgG level, anti-HSV IgM prevalence, and anti-herpesvirus treatment were not associated with the AD or dementia risk among HSV carriers. Lastly, no association was found for the anti-CMV IgG seroprevalence in the full sample or anti-CMV IgG level in the CMV subsample with AD or dementia.

The observed association between the anti-HSV/–HSV-1 IgG prevalence and an increased risk of dementia is in line with the finding of an association of similar magnitude (odds ratio = 2.14) in one case-control study [6], and contradicts the results of one meta-analysis [32] and three serology-based cohort studies [4, 28–29]. The three latter studies had shorter maximum follow-up times than did the present study, which may have limited the ability to capture the phenomenon. They also included younger individuals or individuals with larger age ranges relative to the present cohort. Age is a strong dementia risk factor that is difficult to adjust for adequately and did not confound the present analysis of same-age individuals.

In the present study, the HRs for the non-significant associations between the presence of anti-HSV IgG and the AD risk were of the same magnitude as those for dementia, suggesting that a similar association would have been detectable with greater power, as in a previous cohort study [3]. Furthermore, due to the use of dementia data from medical records and the generally high prevalence of AD among people with dementia [1], most dementia cases in the present cohort were likely AD. In contrast, most previous studies have revealed no association between the anti–HSV-1 IgG [19, 28–31] or anti-HSV IgG [4, 5, 18, 20] prevalence and AD risk, except among *APOE* ɛ4 carriers. Differences in age composition and the anti-HSV/–HSV-1 IgG prevalence between the previous studies and the present work may have influenced the results. This prevalence was higher in three studies [5, 19, 31] and much lower in one study than in the present study [30] and was not reported in another work [29].

In the present study, the non-significant interaction between *APOE* ɛ4 and anti-HSV IgG with regard to the dementia risk was probably due to the lack of power, since the observed effect size was in line with some previous findings [18–20, 25]. This may be due to low proportions of persons with dementia and anti-HSV and anti–HSV-1 IgG negativity. This interaction was significant (*p* < 0.10) in one study in which participants were generally older at the time of inclusion and the dementia incidence was higher than in the present study, which may have improved the analytical power [18]. Two other studies documented interaction between *APOE* ɛ4 and anti-HSV/–HSV-1 IgG with respect to the incidence of AD [19, 20].

The lack of association between the anti-HSV IgM seroprevalence and the AD and dementia risks in the present study is in line with previous findings; one study documented this association only among *APOE* ɛ4 carriers [18] and another revealed an association only between anti-HSV IgG, not IgM, and the AD risk [3]. Anti-HSV IgM was associated with the AD risk in one meta-analysis [17], whereas the evidence was inconclusive in another [32]. In contrast to the present study and one other study [18], the two meta-analyses included studies in which IgM analyses were not stratified by IgG status [3–5] or this information was missing [16], which interferes with comparability and may have resulted in the confounding influence of anti-HSV IgG seropositivity on the findings.

The lack of association between the anti-HSV IgG level and AD or dementia in this study is in line with the findings of another cohort study [29] and two case-control studies [3, 20]. Together with the association between the anti-HSV IgG seroprevalence and dementia, this finding indicates that the presence, rather than level, of IgG is indicative of a dementia risk. This finding is in line with a previous finding on similar anti-HSV IgG titers among persons with frequent and infrequent symptoms of orofacial HSV reactivations [50] indicating that anti-HSV IgG level may not be a reliable indicator of propensity for reactivation. However, potential interactions between IgG level and *APOE* ɛ4, found in a previous study [18], could not be tested due to sample size restrictions.

The lack of association between anti-herpesvirus treatment and the risk of AD or dementia among anti-HSV IgG seropositive individuals contrasts with the findings of a population-based nested case-control study [34]. Total prescription frequencies were similar in the two studies, but *APOE* ɛ4 carriage in the anti-HSV IgG group was more prevalent in the previous study than in the present work [34], which may have interfered with the comparability of the findings. Persons diagnosed with HSV when seeking care for HSV symptoms or self-reported reactivation had a reduced risk of dementia with anti-herpesvirus treatment in most previous register-based studies [16, 35–40], except one [39]. Schnier et al. [40] obtained heterogenous results in a study of four registers; data from two registers showed that anti-HSV drugs reduced the risk of dementia slightly, whereas data from the other two registers showed no such effect. The use of HSV diagnosis and self-reported reactivation differs from the examination of seropositivity, as in the present work, in that only symptomatic individuals are studied, whereas most seropositive individuals do not have symptomatic reactivation. Symptomatic reactivation may be overrepresented among *APOE* ɛ4 carriers and these factors may interfere with risk assessment [22–24]. Further, observational studies cannot adequately account for any bias introduced by physicians or patients regarding who is prescribed a drug and for which indications. The effects of anti-herpesvirus treatment on the dementia risk need to be investigated in randomized controlled trials to adequately circumvent these issues.

The mixed findings of previous studies of associations between anti-CMV IgG with AD or dementia risk highlight the need for further study of these potential relationships [6, 10–12, 32, 41–43]. Moreover, we detected no interaction between anti-CMV IgG and anti-HSV IgG seroprevalence with respect to the dementia risk, in contrast to the findings of a case-control study of the AD risk [41]. That study had a higher proportion of women (75%), younger mean age (61 years), and earlier timing of plasma sampling (beginning in 1986) than did the present study [41], which may compromise the comparability of the results, especially because anti-herpesvirus treatment became more common in later decades.

The strengths of the present study were the examination of a population-based contemporary cohort over a 15-year follow-up period, the very low proportion of missing data, the rigorous serological assessments performed, and the thorough medical records review on which the AD and dementia diagnoses and anti-herpesvirus treatment data were based. Additionally, the difficult-to-handle influence of age, the strongest risk factor for AD and dementia, was eliminated by including individuals only months from their 70^th^ birthday. The limitations of this study include the underrepresentation of persons with diabetes, congestive heart failure, and stroke in the PIVUS cohort, as reported previously [44], and the lack of information on treatment compliance, dosage, and length and the number of prescriptions, which prevented the analysis of dose dependency in examining the associations of interest. Statistical power was limited in some analyses. Dementia cases may be underreported, since the dementia data collection relied on medical records; however, publicly funded health care in Sweden and the generally high educational attainment of the PIVUS population may have improved the chances of participants seeking medical attention for issues such as memory impairment. The 15-year long follow-up ensures that also individuals who were diagnosed in later dementia stages were detected. Lastly, some cases of Alzheimer’s disease may have been misclassified as vascular dementia or other, as the diagnoses were made during routine clinical practice and not all patients were evaluated extensively in specialized memory clinics.

### Conclusion

In this prospective epidemiological study, anti-HSV IgG seroprevalence was associated with a doubled risk of dementia in an older population. This finding adds to the evidence supporting a role of HSV in dementia. No such effect was found for CMV. The low cumulative incidence of AD may have impaired the statistical power to detect associations. HSV did not appear to interact with CMV or *APOE* ɛ4 carriage in association with AD or dementia, but further studies with more power are needed to examine such potential interactions. The potential effect of anti-herpesvirus treatment on the dementia risk needs to be studied in randomized controlled trials to circumvent issues inherent to observational studies.

## CREDIT AUTHOR STATEMENT

Bodil Weidung (Conceptualization; Data curation; Formal analysis; Funding acquisition; Investigation; Methodology; Project administration; Software; Supervision; Visualization; Writing – review & editing); Erika Vestin (Formal analysis; Investigation; Visualization; Writing – original draft); Gustaf Boström (Data curation; Supervision; Writing – review & editing); Jan Olsson (Data curation; Methodology; Resources; Supervision; Validation; Writing – review & editing); Fredrik Elgh (Conceptualization; Resources; Supervision; Validation; Writing – review & editing); Lars Lind (Conceptualization; Data curation; Funding acquisition; Investigation; Methodology; Project administration; Resources; Writing – review & editing); Lena Kilander (Data curation; Investigation; Resources; Writing – review & editing); Hugo Lövheim (Conceptualization; Methodology; Project administration; Supervision; Validation; Writing – review & editing).

## Supplementary Material

Supplementary Material

## Data Availability

The data supporting the findings of this study are available on reasonable request from the corresponding author. The data are not publicly available due to privacy or ethical restrictions.
