# Supplementary material for: Herpes Simplex Viral Infection Doubles the Risk of Dementia in a Contemporary Cohort of Older Adults: A Prospective Study
Source: J Alzheimers Dis. 2024 Feb 13;97(4):1841–50. doi: 10.3233/JAD-230718 (PMC10894565; doi:10.3233/JAD-230718)
Supplement: Supplementary Material [file jad-97-jad230718-s001.pdf]

Supplementary Material

Herpes Simplex Viral Infection Doubles the Risk of Dementia in a Contemporary Cohort of Older Adults: A Prospective Study

SUPPLEMENTARY RESULTS

Larger proportions of participants included than not included in the HSV and HSV-1 subsamples had ≤9 years education (HSV: 58.5% versus 49.2%, $p = 0.022$; HSV-1: 59.3% vs. 49.8%, $p = 0.008$) and anti-CMV IgG positivity (HSV: 80.5% versus 68.2%, $p < 0.001$; HSV-1: 81.4% versus 69.1%, $p < 0.001$). Relative to other participants, the CMV subsample was characterized by a larger proportion of females (52.2% versus 42.7%, $p = 0.013$), larger proportion of persons with ≤9 years education (58.7% versus 50.2%, $p = 0.024$), and greater prevalence of anti-HSV IgM positivity (9.2% versus 4.5%, $p = 0.026$).

Anti–HSV-1 IgG positivity nearly doubled the risk of dementia in the basic [hazard ratio (HR) = 1.92, $p = 0.034$] and fully adjusted (HR = 1.95, $p = 0.031$) models (Supplementary Table S1, Supplementary Figure 1). No significant association between anti–HSV-1 IgG positivity and AD was found in the full sample (Supplementary Table 1). The interaction between anti–HSV-1 IgG and *APOE* ε4 positivity was not significant (Supplementary Table 1).

The results of proportional hazard assumption testing are presented here, including numerical (Supplementary Tables 2-4) and graphical tests (Supplementary Figures 2.1.1-4.2.2). In the graph legends: 0 = no and 1 = yes.

Supplementary Table 1. HRs for AD and dementia with anti-HSV type 1 IgG positivity and its interaction with *APOE* ε4 positivity for the full sample.

	AD		Dementia					
	Basic model		Basic model		Fully adjusted model		Interaction model	
	HR (95% CI)	*p*	HR (95% CI)	*p*	HR (95% CI)	*p*	HR (95% CI)	*p*
Anti–HSV-1 IgG pos	2.12 (0.88–5.10)	0.093	1.92 (1.05–3.51)	0.034	1.95 (1.06–3.57)	0.031	1.69 (0.78–3.65)	0.182
Sex (women)	1.31 (0.68–2.54)	0.424	0.91 (0.57–1.45)	0.691	0.89 (0.56–1.42)	0.623	0.88 (.55–1.41)	0.603
Education ≥10 y					0.84 (0.52–1.36)	0.474	0.84 (0.52–1.37)	0.490
APOE ε4 pos					1.75 (1.09–2.81)	0.021	1.32 (0.43–4.05)	0.623
Anti–HSV-1 IgG pos x *APOE* ε4 pos							1.41 (0.41–4.85)	0.588

Data were obtained with Cox proportional-hazards regression models.
HR, hazard ratio; AD, Alzheimer's disease; CI, confidence interval; HSV-1, herpes simplex virus type 1; IgG, immunoglobulin G; pos, positive; *APOE* ε4, apolipoprotein E4.

Supplementary Table 2. *p* for Shoenfeld test of the full sample model.

Partial residual	*p* for Pearson correlation with ranked time			
	AD, basic	Dementia, basic	Dementia, fully	Dementia, interaction
**Anti-HSV IgG pos model**				
Anti-HSV IgG pos	0.830	0.941	0.952	0.949
Sex (women)	0.372	0.190	0.195	0.197
Education ≥10 y			0.876	0.878
APOE ε4 pos			0.118	0.111
Anti-HSV IgG pos x *APOE* ε4 pos				0.208
**Anti-HSV-1 IgG pos model**				
Anti-HSV-1 IgG pos	0.551	0.894	0.894	0.889
Sex (women)	0.370	0.189	0.192	0.192
Education ≥10 y			0.876	0.879
APOE ε4 pos			0.122	0.119
Anti-HSV-1 IgG pos x *APOE* ε4 pos				0.241
**Anti-CMV IgG pos model with HSV**				
Anti-CMV IgG pos	0.923	0.452	0.459	0.449
Sex (women)	0.368	0.187	0.193	0.194
Education ≥10 y			0.869	0.868
Anti-HSV IgG pos			0.942	0.942
Anti-CMV IgG pos x anti-HSV IgG pos				0.462
**Anti-CMV IgG pos model with HSV-1**				
Anti-CMV IgG pos	0.923	0.452	0.457	0.444
Sex (women)	0.368	0.187	0.191	0.194
Education ≥10 y			0.867	0.863
Anti-HSV-1 IgG pos			0.894	0.893
Anti-CMV IgG pos x anti-HSV-1 IgG pos				0.305

AD, Alzheimer's disease; HSV, herpes simplex virus; IgG, immunoglobulin G; pos, positive; *APOE* ε4, apolipoprotein E4; HSV-1, herpes simplex virus type 1; CMV, cytomegalovirus.

Supplementary Table 3. *p* for Shoenfeld test of the HSV subsample model.

	p for Pearson correlation with ranked time		
Partial residual	AD, basic	Dementia, basic	Dementia, fully
**Anti-HSV IgM pos model**			
Anti-HSV IgM pos	0.593	0.807	0.810
Sex (women)	0.1.66	0.112	0.118
Education ≥10 y			0.656
APOE ε4 pos			0.181
**Anti-HSV-IgG level model**			
Anti-HSV- IgG level	0.232	0.858	0.886
Sex (women)	0.165	0.112	0.118
Education ≥10 y			0.656
APOE ε4 pos			0.181
**Anti-herpesvirus drugs 5 y**			
Anti-herpesvirus drugs 5 y	0.335	0.340	0.315
Sex (women)	0.167	0.114	0.121
Education ≥10 y			0.651
APOE ε4 pos			0.176

AD, Alzheimer's disease; HSV, herpes simplex virus; IgG, immunoglobulin G; pos, positive; *APOE* ε4, apolipoprotein E4.

Supplementary Table 4. *p* for Shoenfeld test of the HSV-1 subsample model.

	p for Pearson correlation with ranked time		
Partial residual	AD, basic	Dementia, basic	Dementia, fully
**Anti-HSV IgM pos model**			
Anti-HSV IgM pos	0.411	0.687	0.693
Sex (women)	0.163	0.073	0.074
Education ≥10 y			0.762
APOE ε4 pos			0.235
**Anti-HSV-IgG level model**			
Anti-HSV- IgG level	0.242	0.931	0.903
Sex (women)	0.163	0.073	0.074
Education ≥10 y			0.765
APOE ε4 pos			0.235
**Anti-herpesvirus drugs 5 y**			
Anti-herpesvirus drugs 5 y	0.336	0.401	0.389
Sex (women)	0.164	0.073	0.075
Education ≥10 y			0.764
APOE ε4 pos			0.231

AD, Alzheimer's disease; HSV-1, herpes simplex virus type 1; IgG, immunoglobulin G; pos, positive; *APOE* ε4, apolipoprotein E4.

Supplementary Figure 1. Incident dementia according to anti-HSV type 1 IgG positivity.

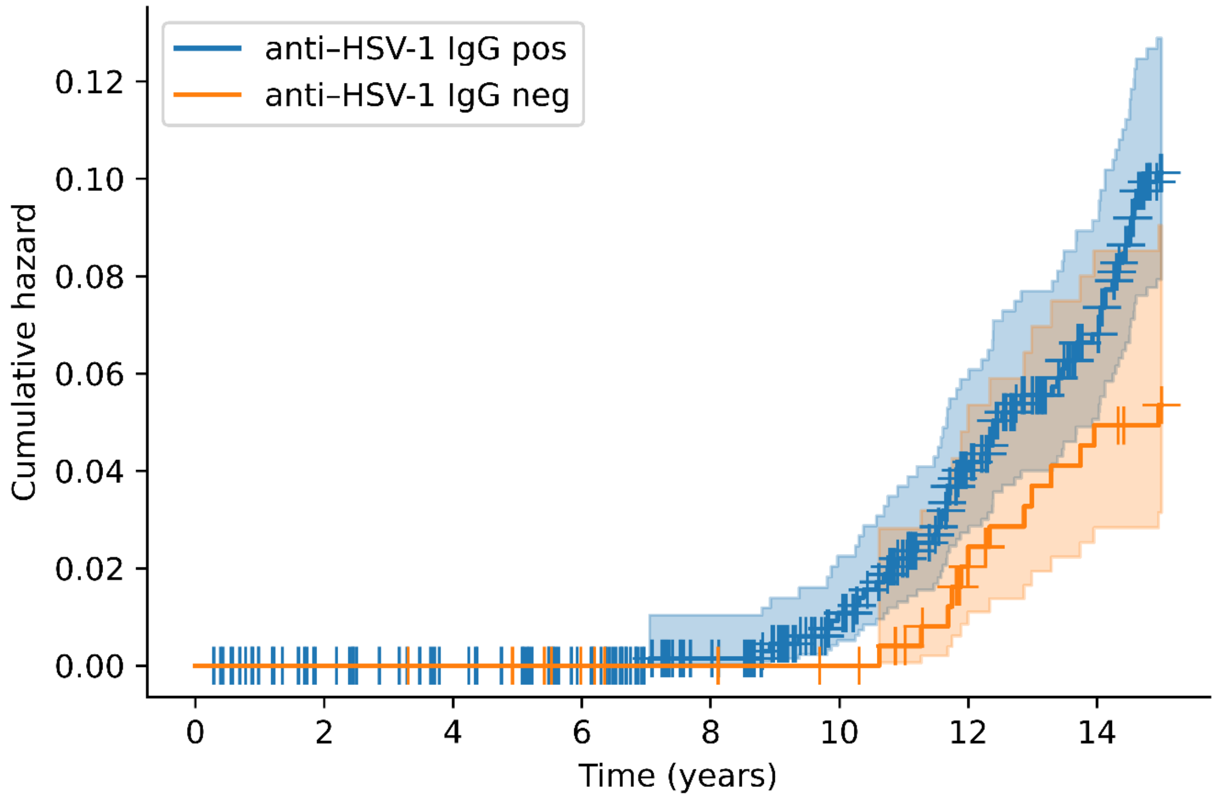

Kaplan–Meier cumulative hazard of incident dementia according to anti-HSV type 1 IgG positivity. Each + denotes a censored observation. HSV, herpes simplex virus; IgG, immunoglobulin G.

Supplementary Figure 2.1 AD outcome in the full sample

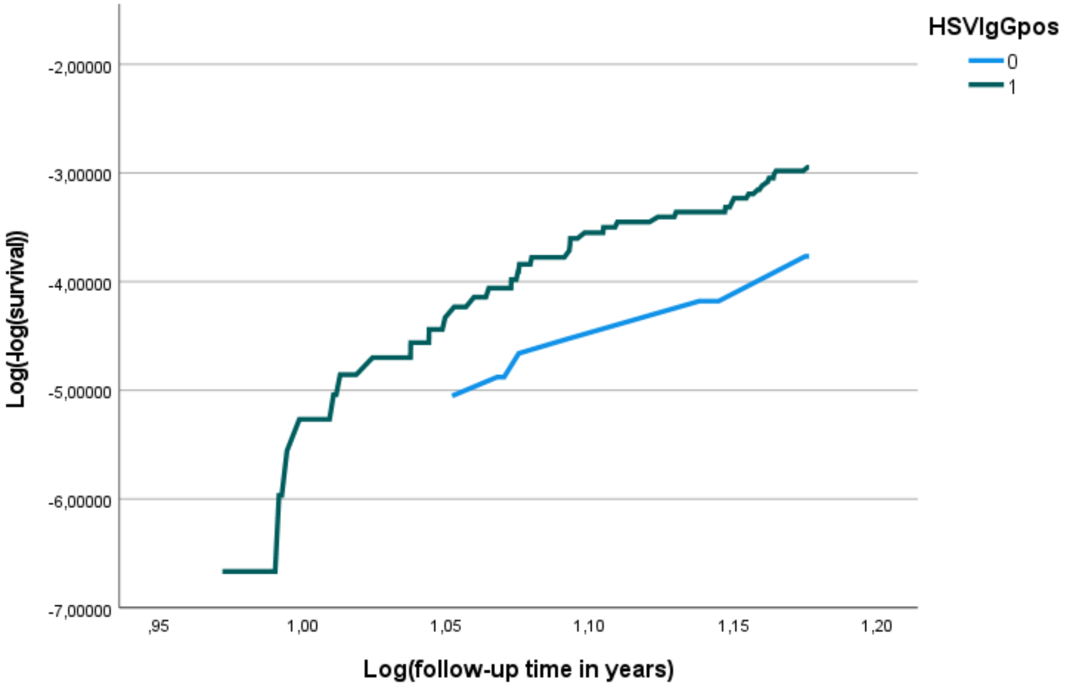

Supplementary Figure 2.1.1

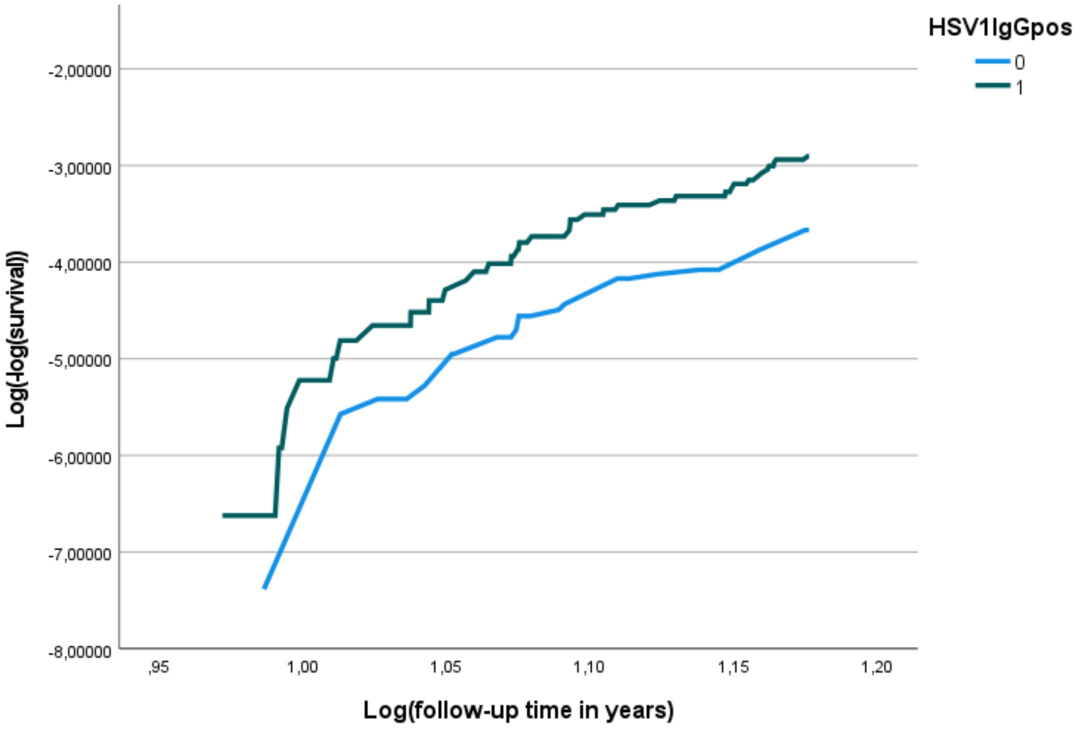

Supplementary Figure 2.1.2

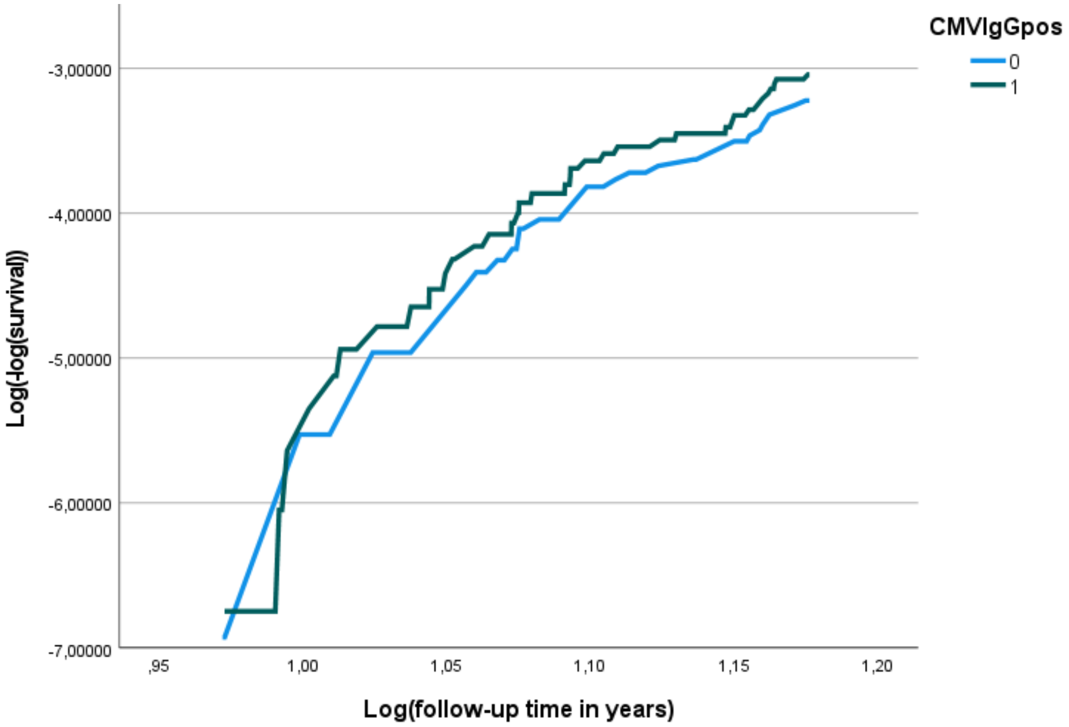

Supplementary Figure 2.1.3

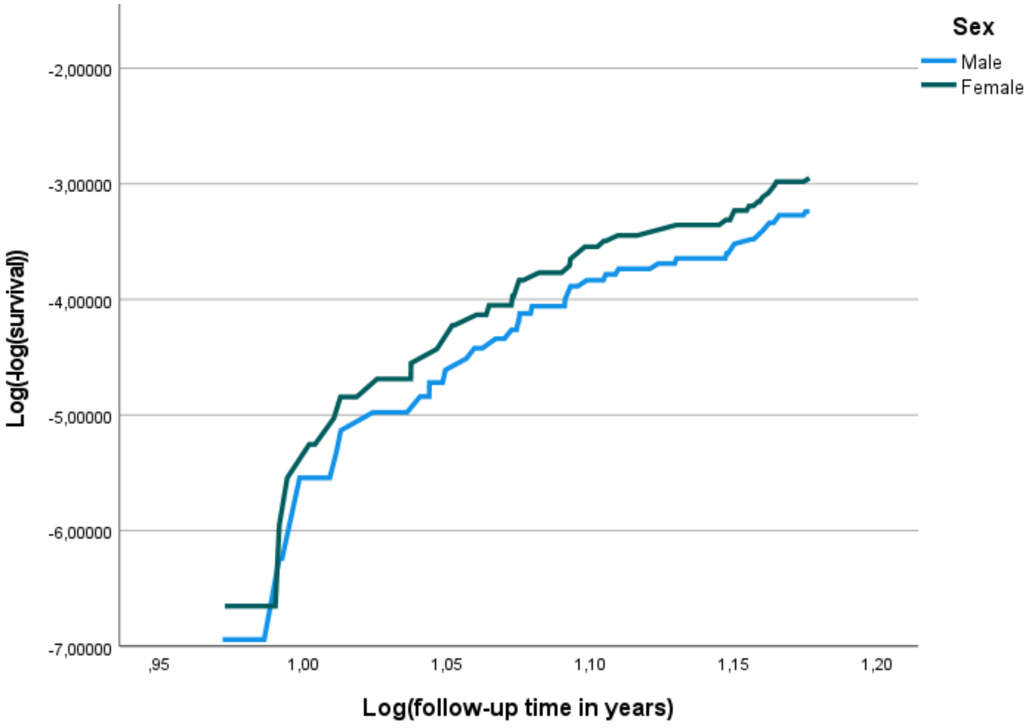

Supplementary Figure 2.1.4

Supplementary Figure 2.2 Dementia outcome in the full sample

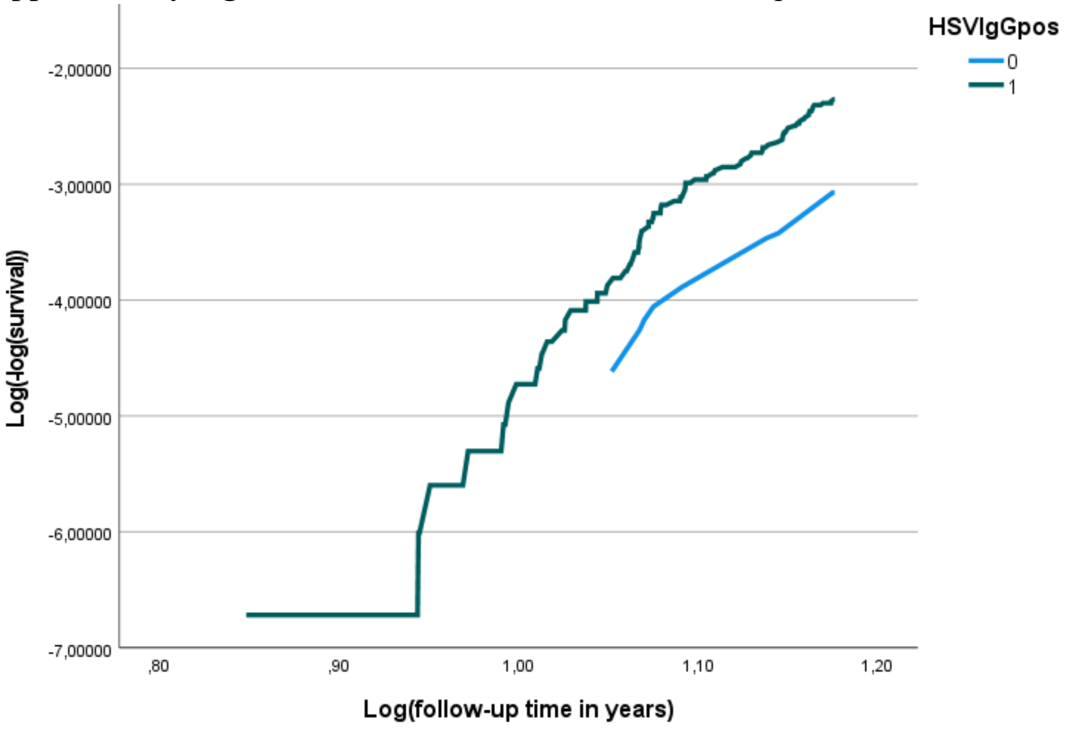

Supplementary Figure 2.2.1

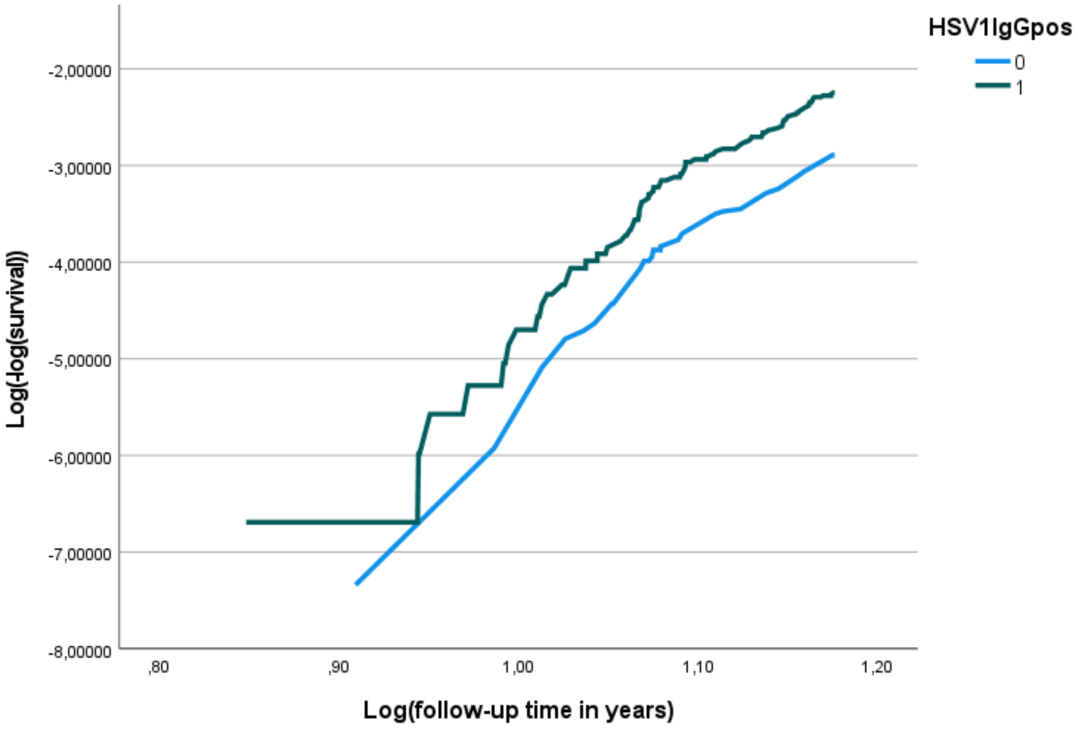

Supplementary Figure 2.2.2

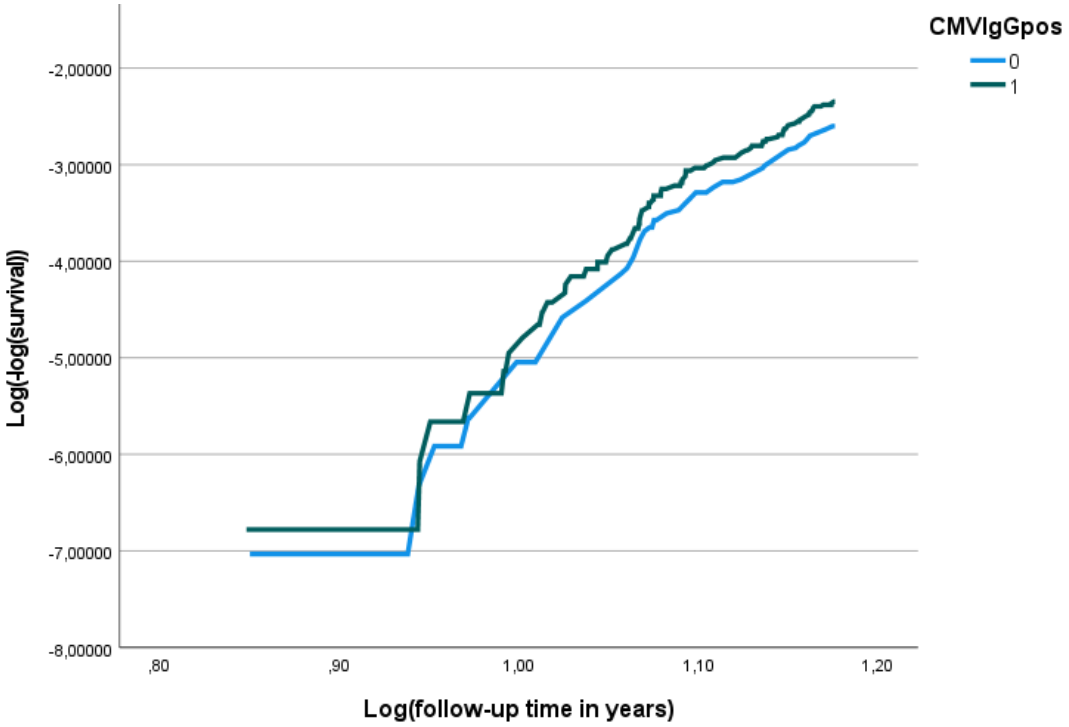

Supplementary Figure 2.2.3

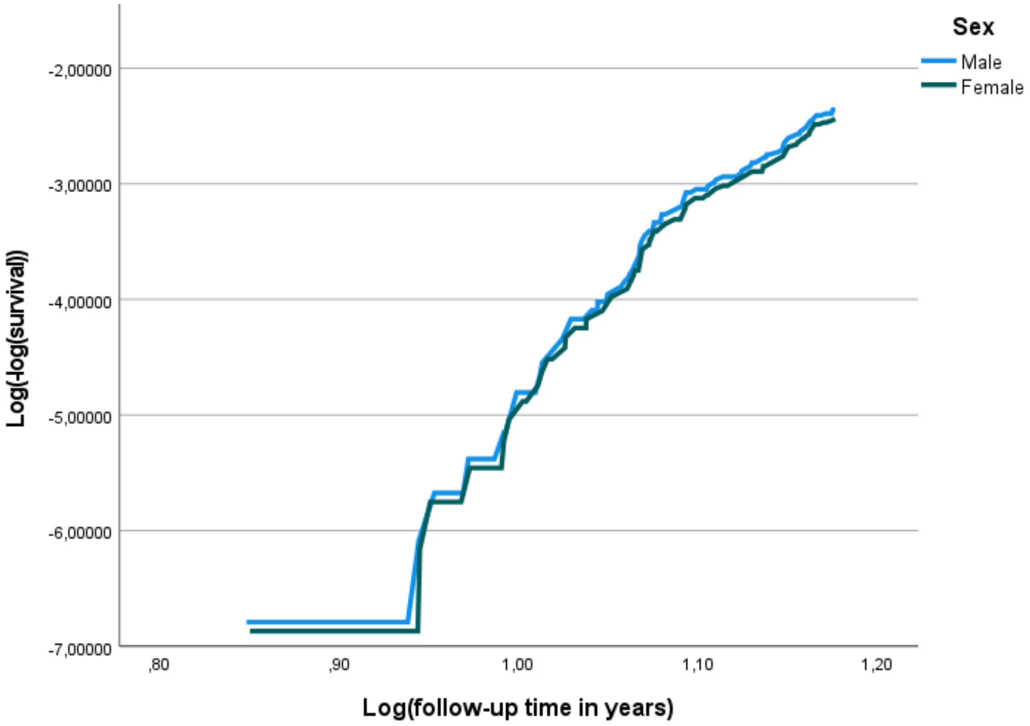

Supplementary Figure 2.2.4

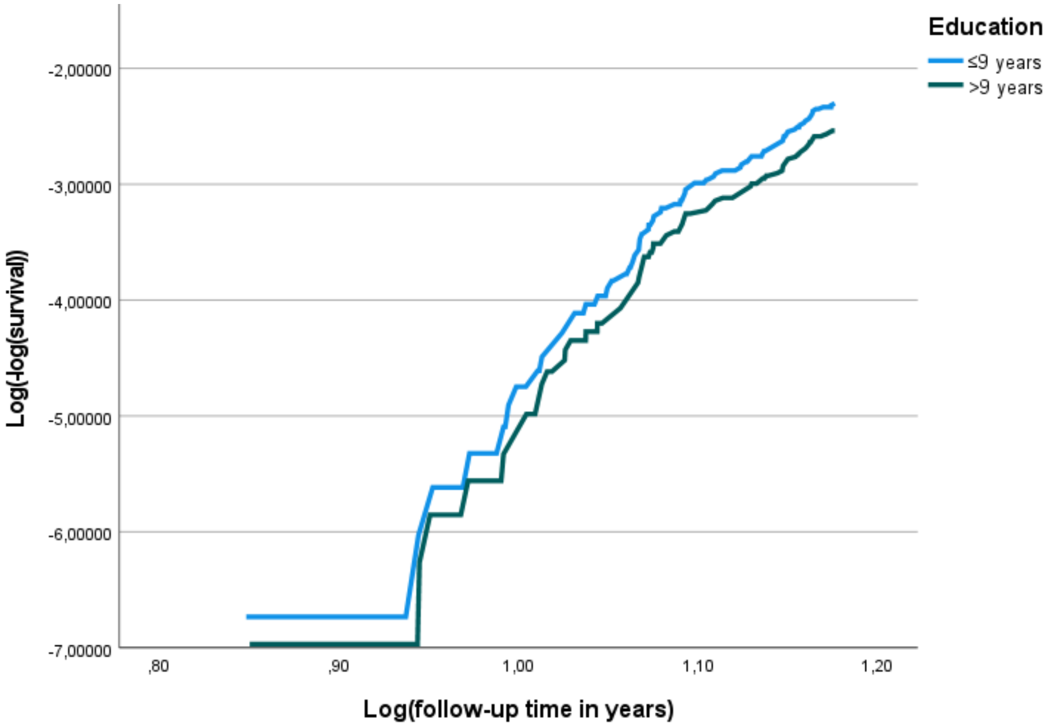

Supplementary Figure 2.2.5

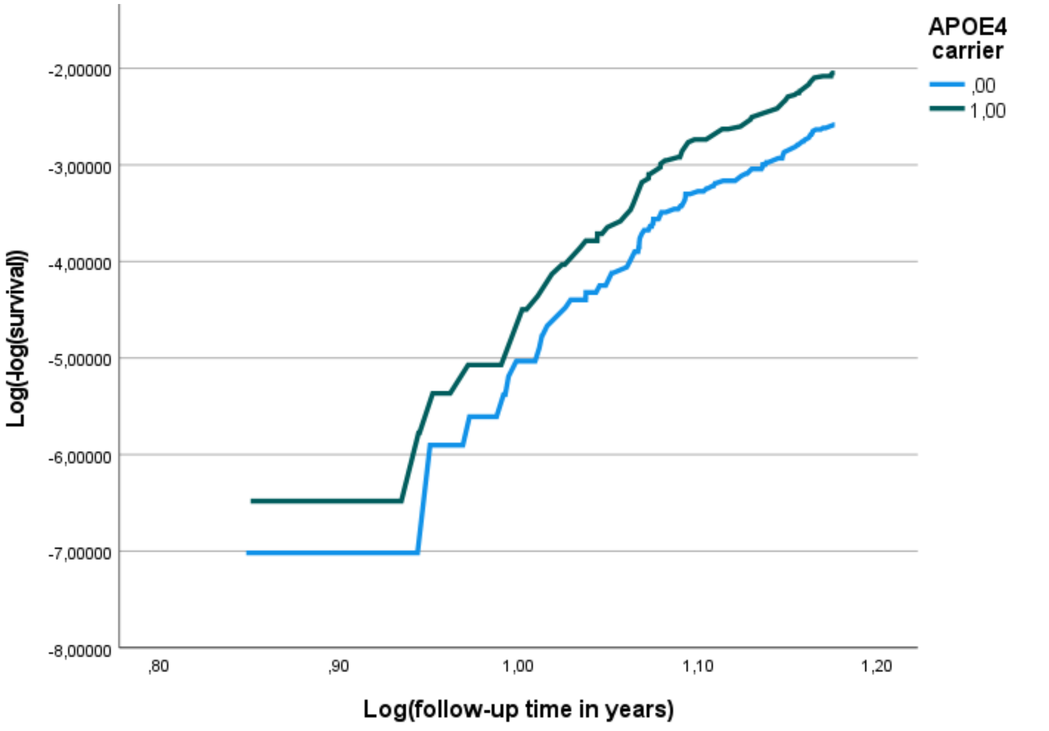

Supplementary Figure 2.2.6

Supplementary Figure 3.1 AD outcome in HSV subsample

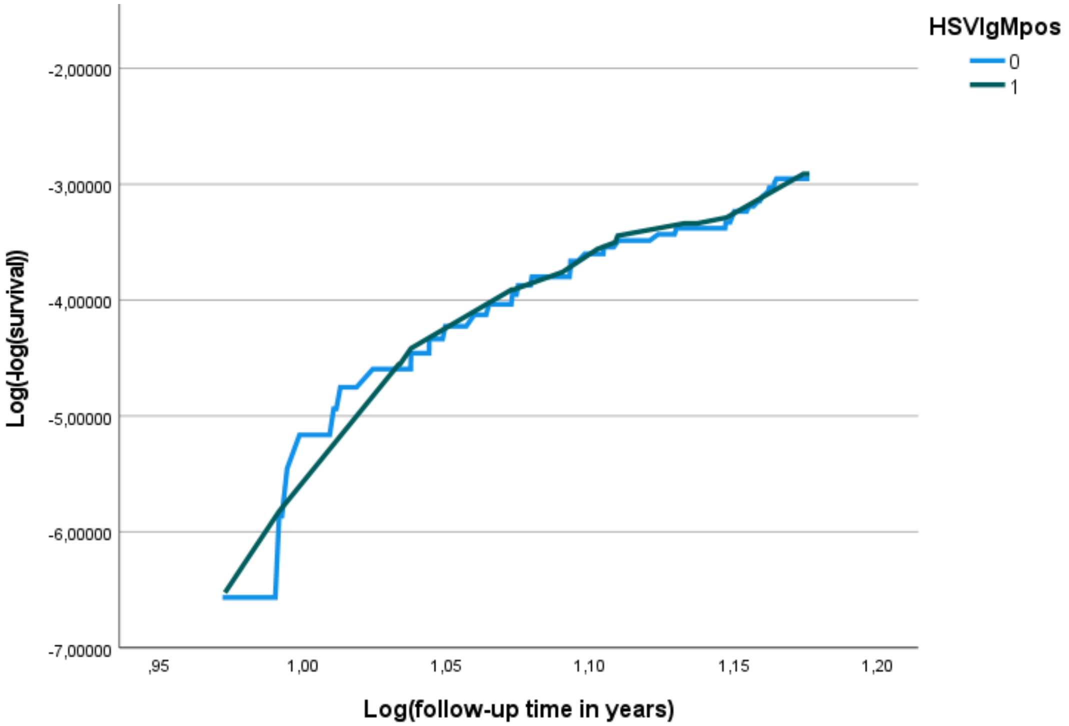

Supplementary Figure 3.1.1

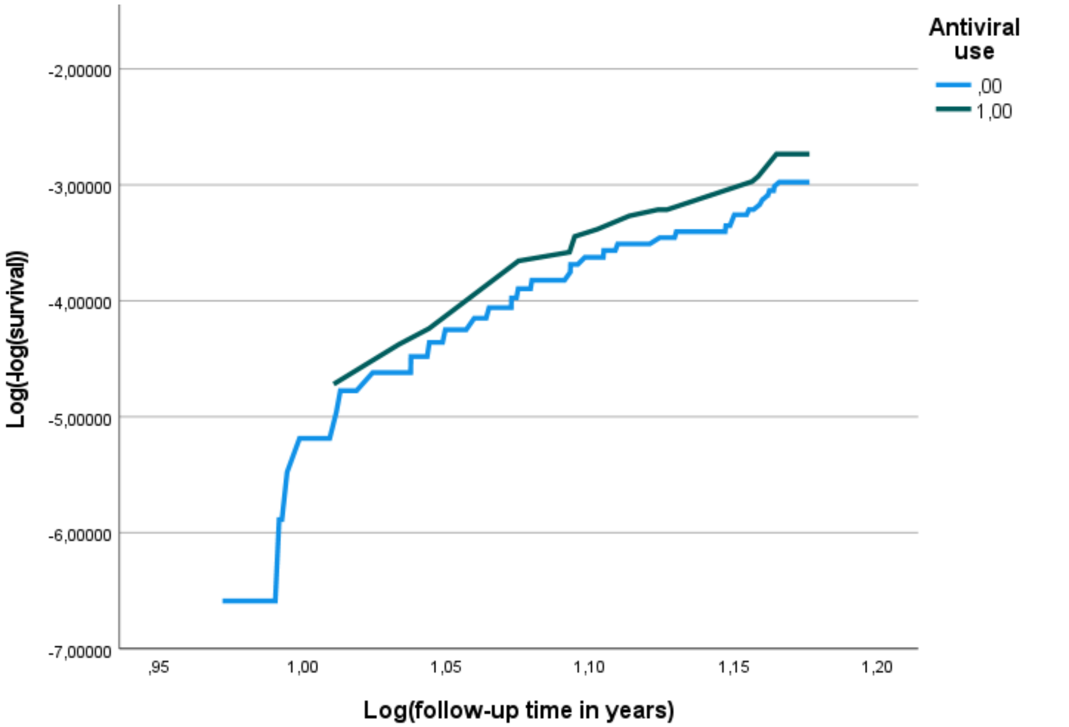

Supplementary Figure 3.1.2

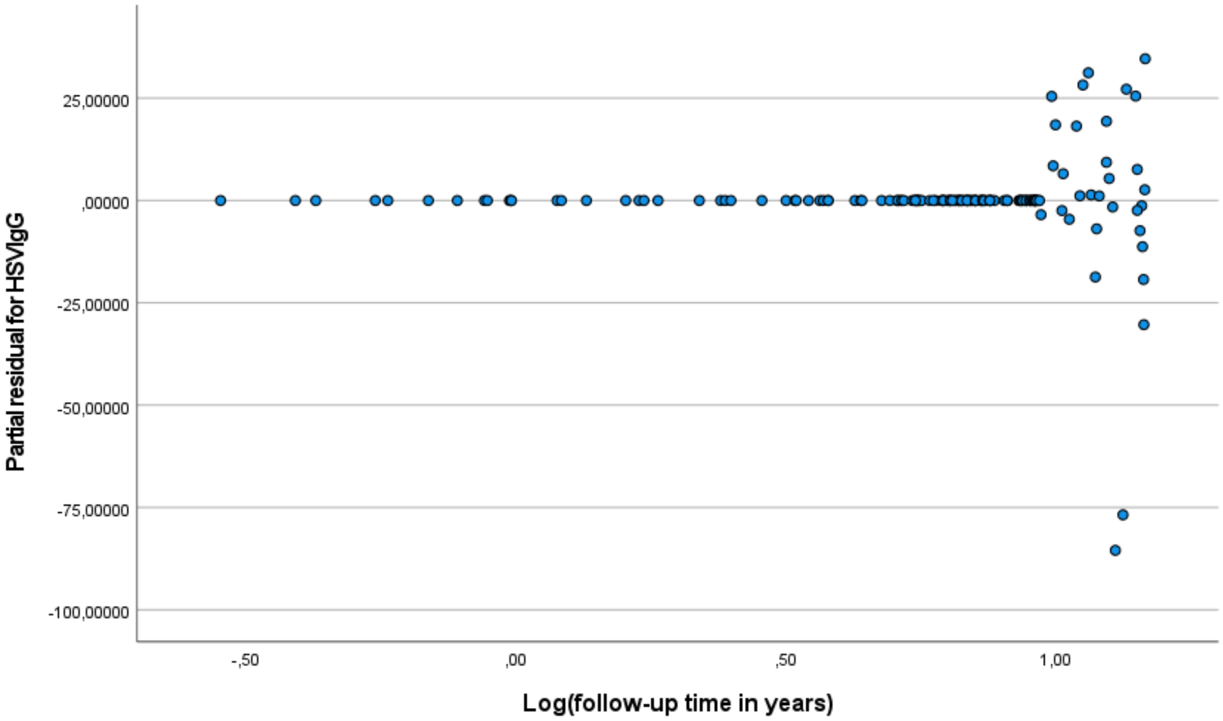

Supplementary Figure 3.1.3

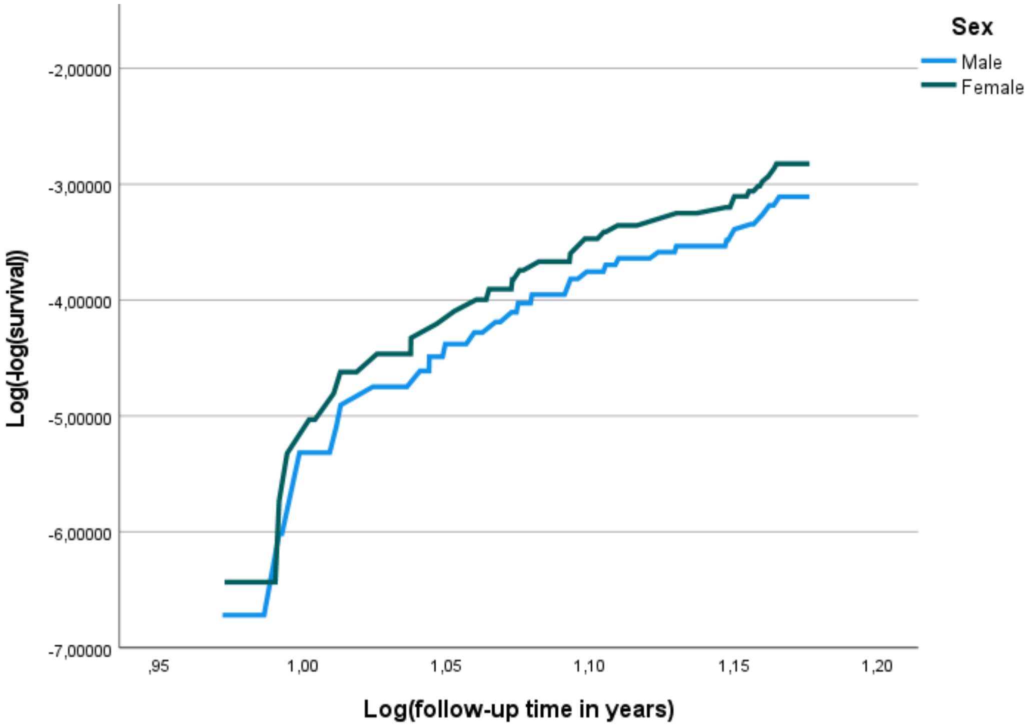

Supplementary Figure 3.1.4

Supplementary Figure 3.2 Dementia outcome in HSV subsample

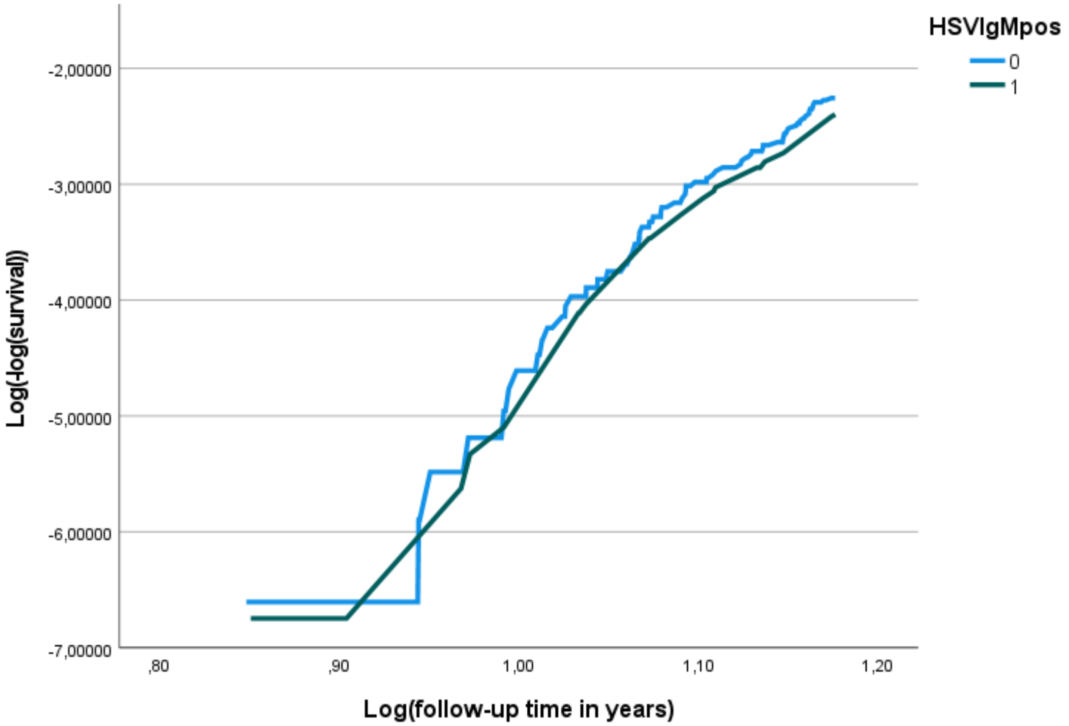

Supplementary Figure 3.2.1

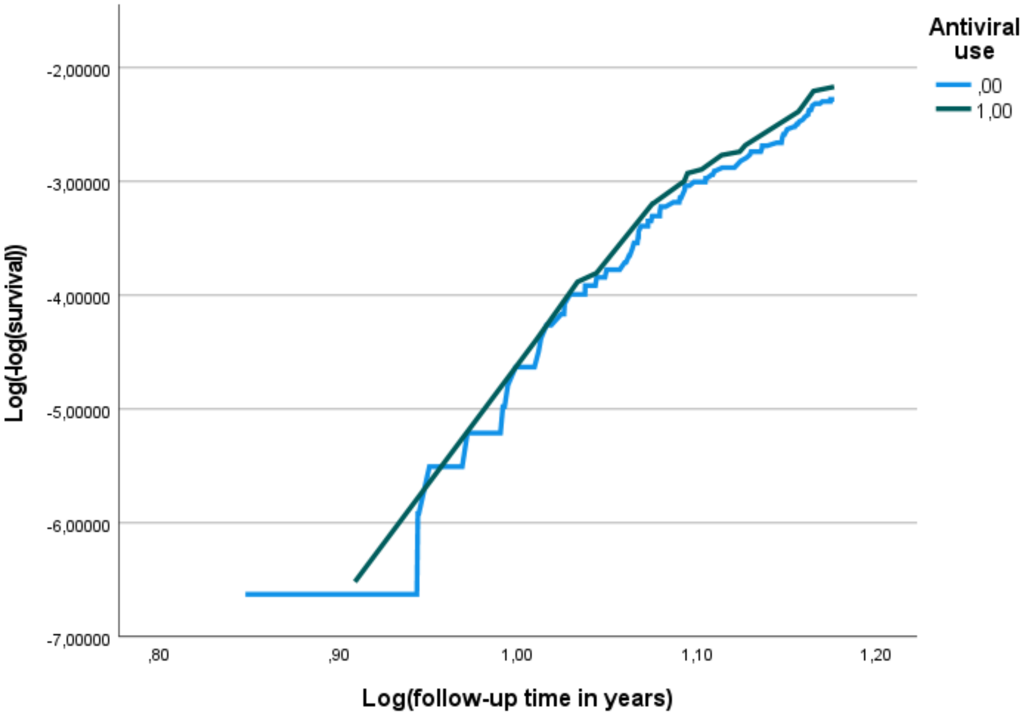

Supplementary Figure 3.2.2

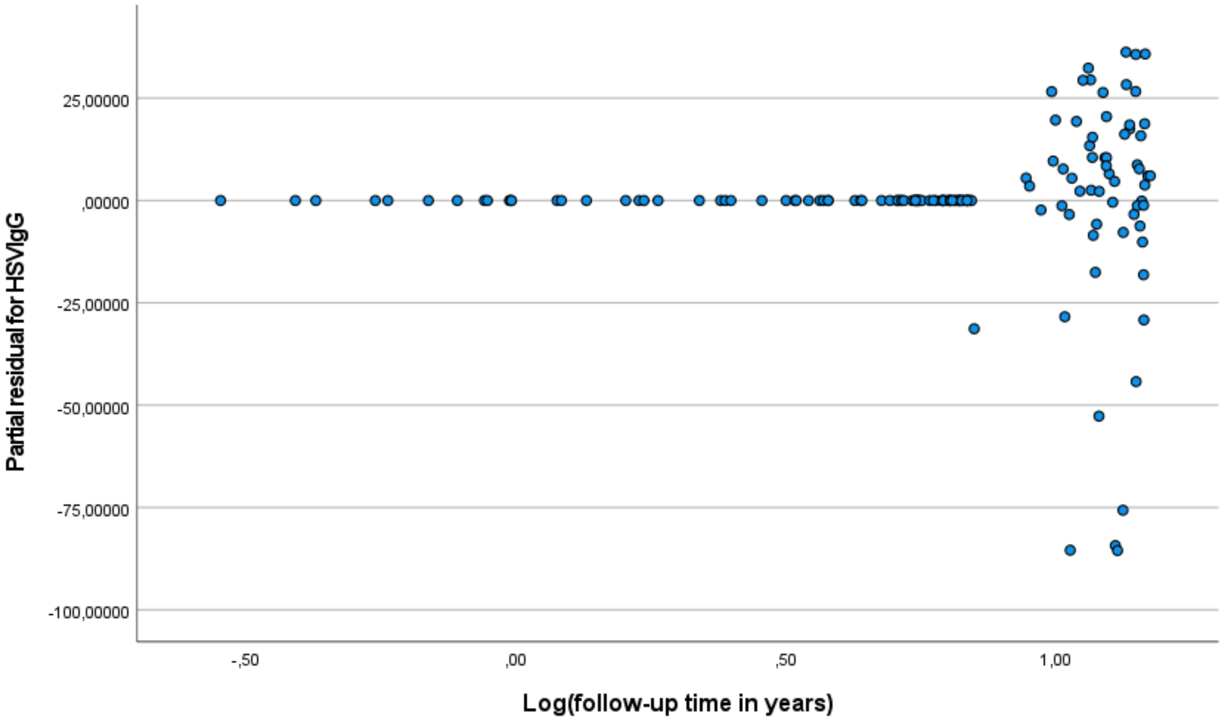

Supplementary Figure 3.2.3

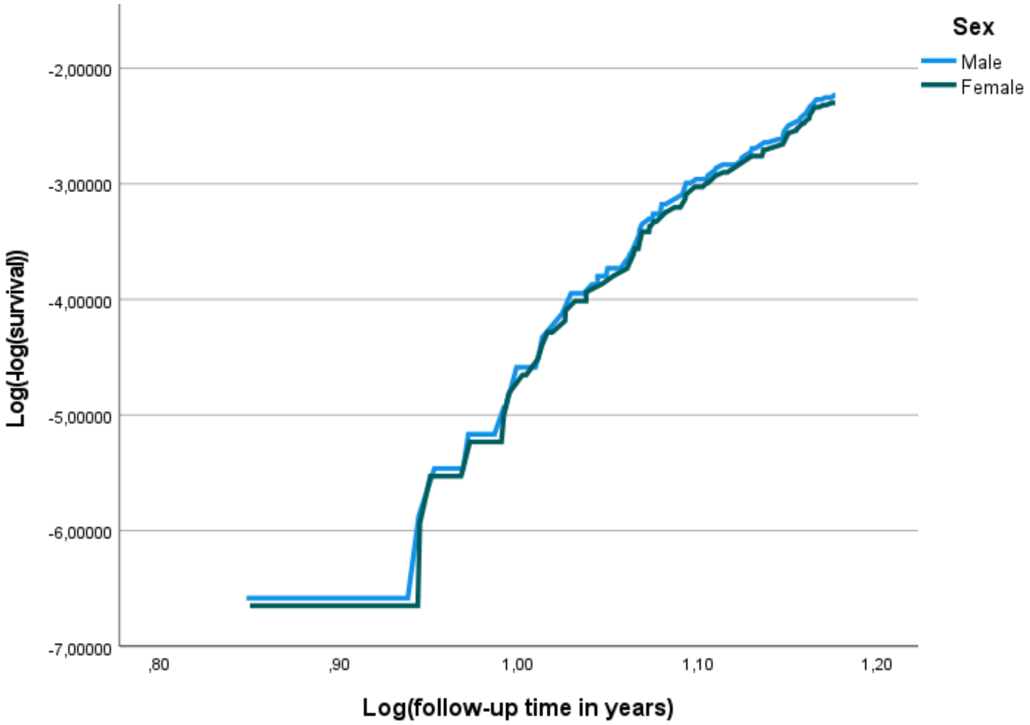

Supplementary Figure 3.2.4

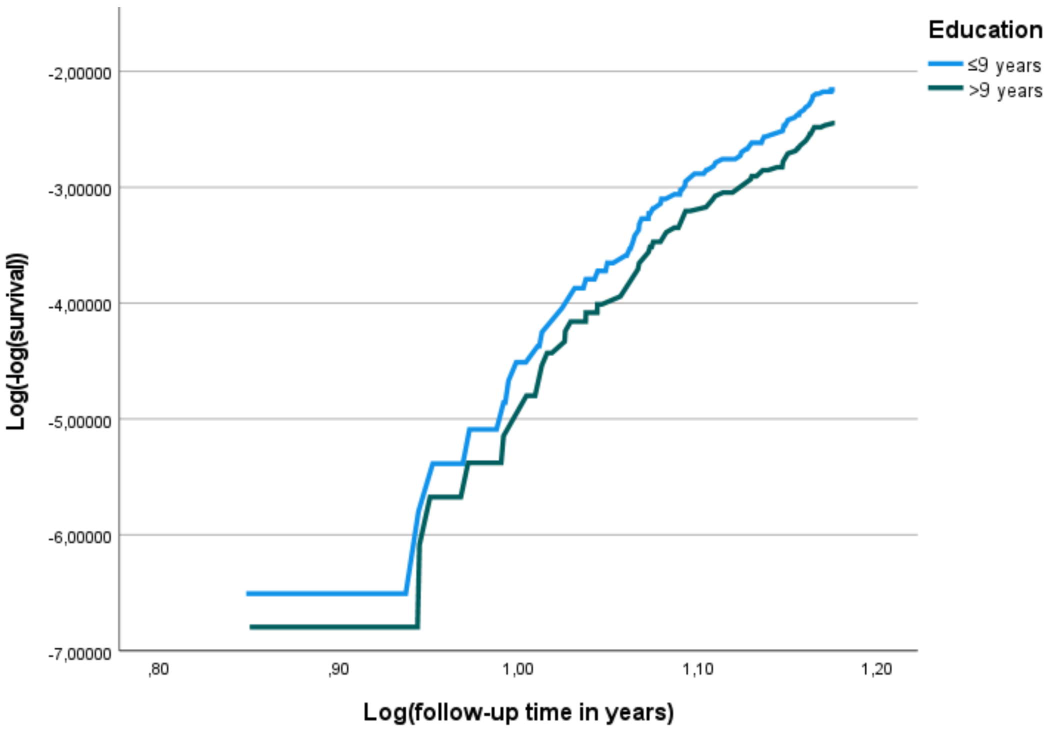

Supplementary Figure 3.2.5

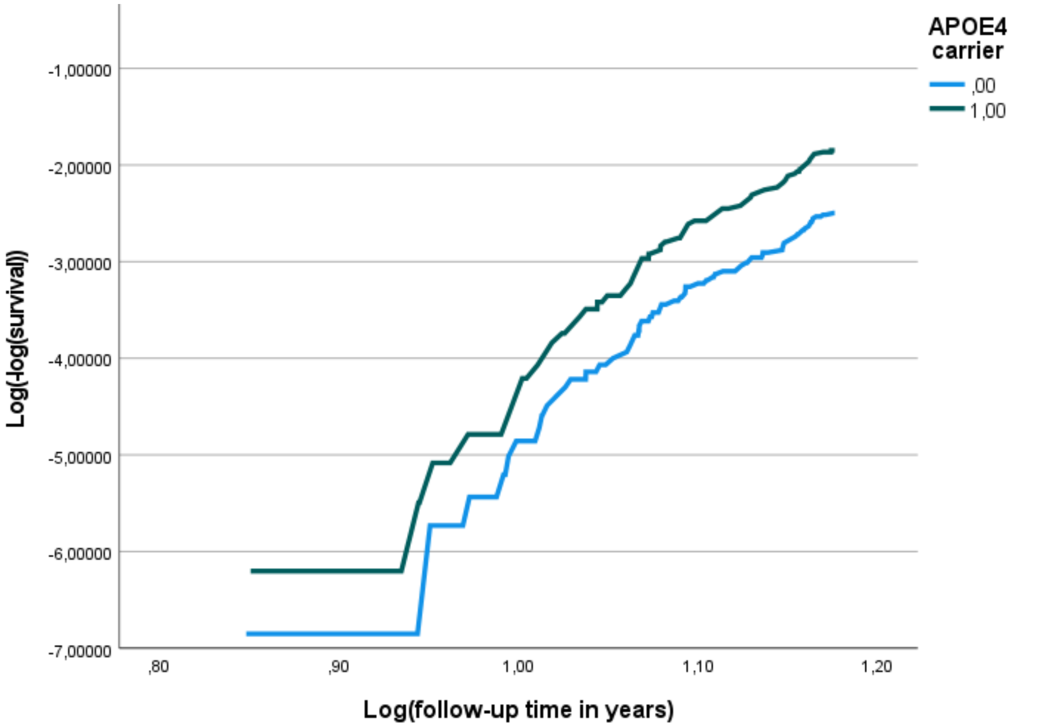

Supplementary Figure 3.2.6

Supplementary Figure 4.1 Dementia outcome in CMV subsample

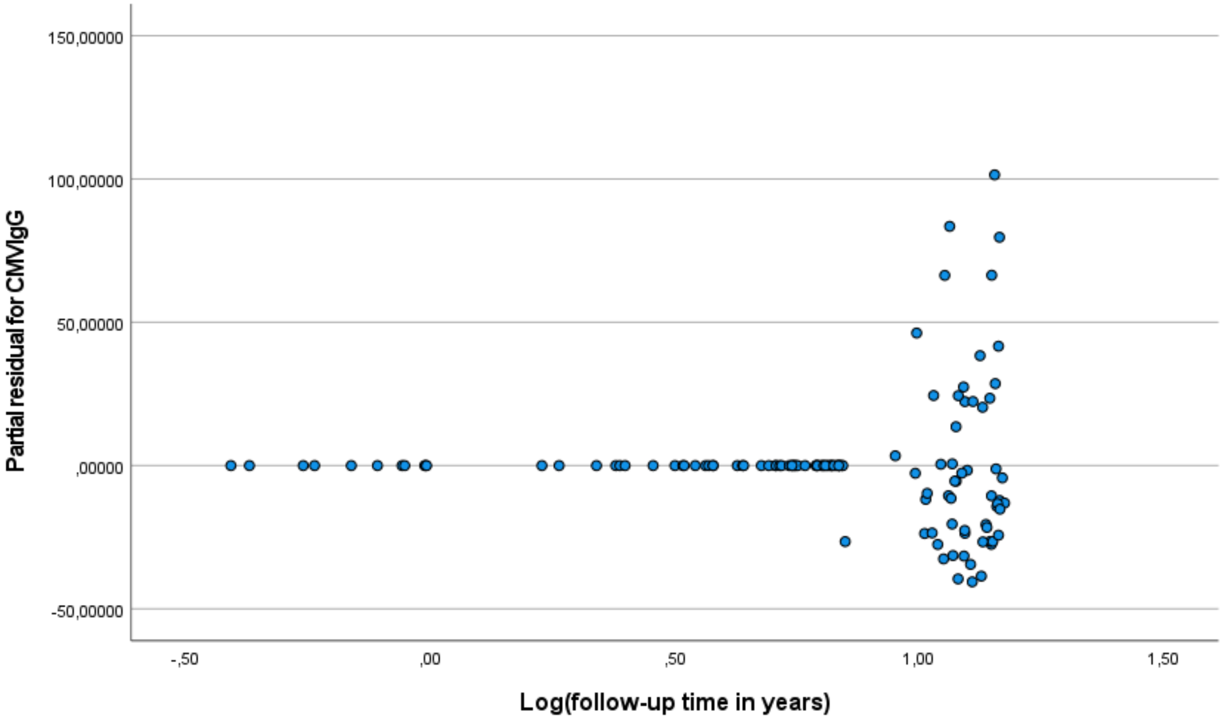

Supplementary Figure 4.1.1

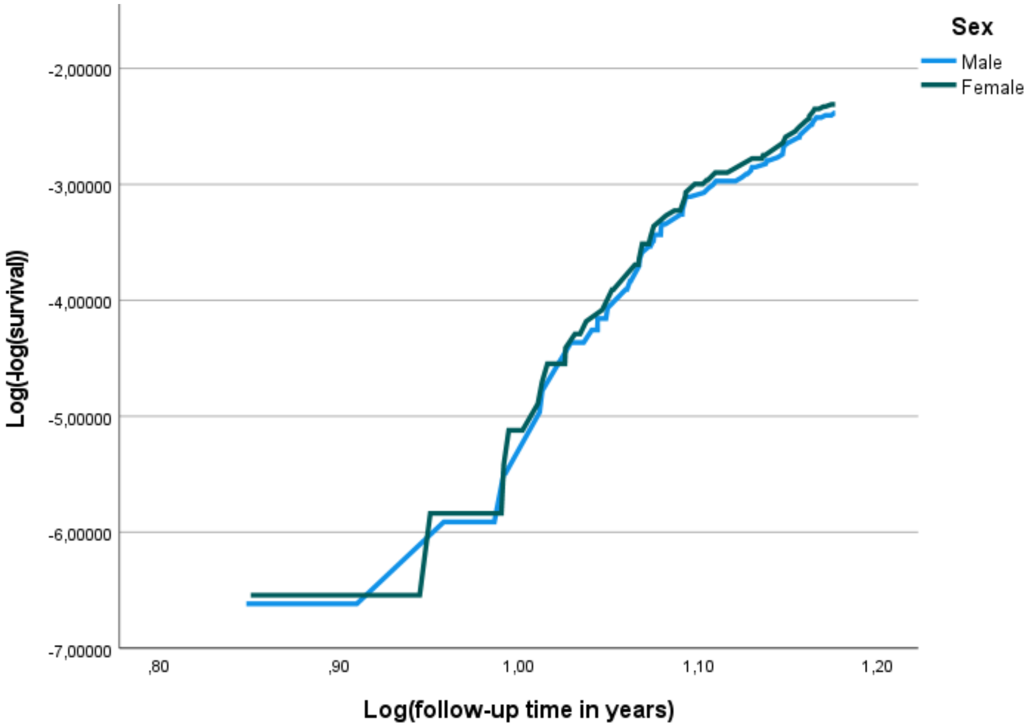

Supplementary Figure 4.1.2

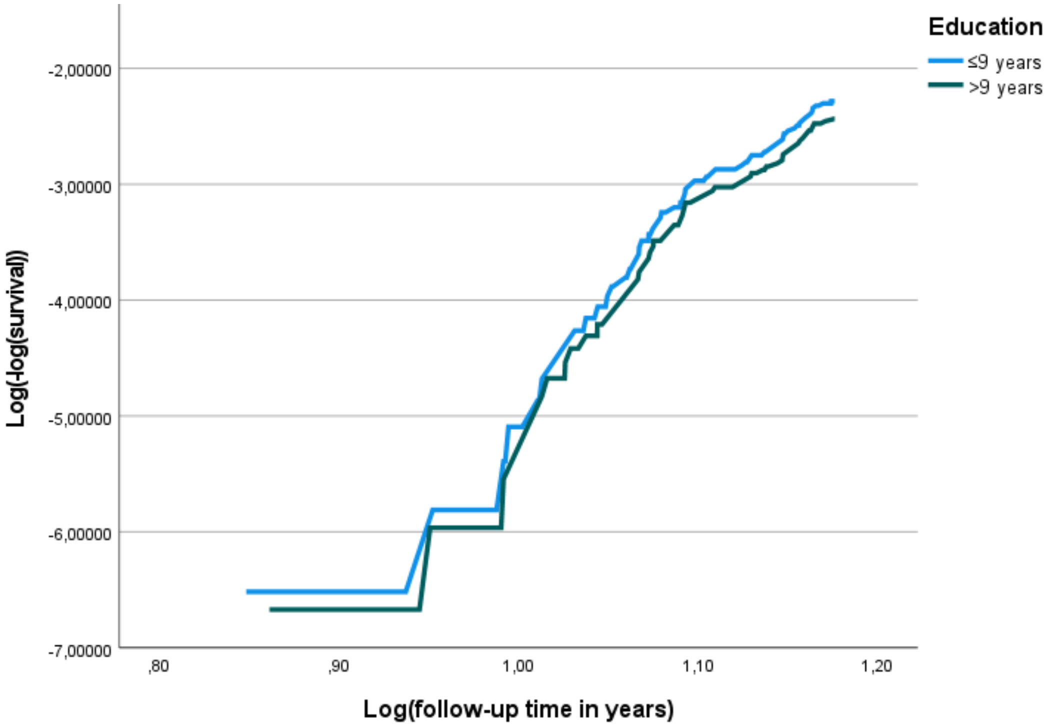

Supplementary Figure 4.1.3

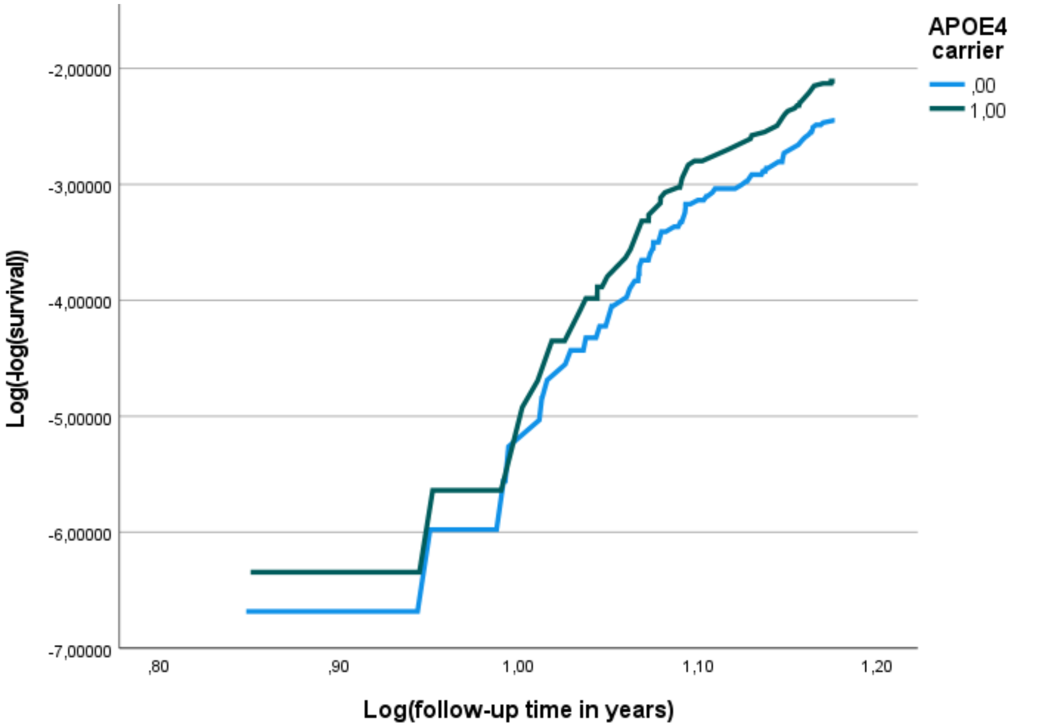

Supplementary Figure 4.1.4

Supplementary Figure 4.2 AD outcome in CMV subsample

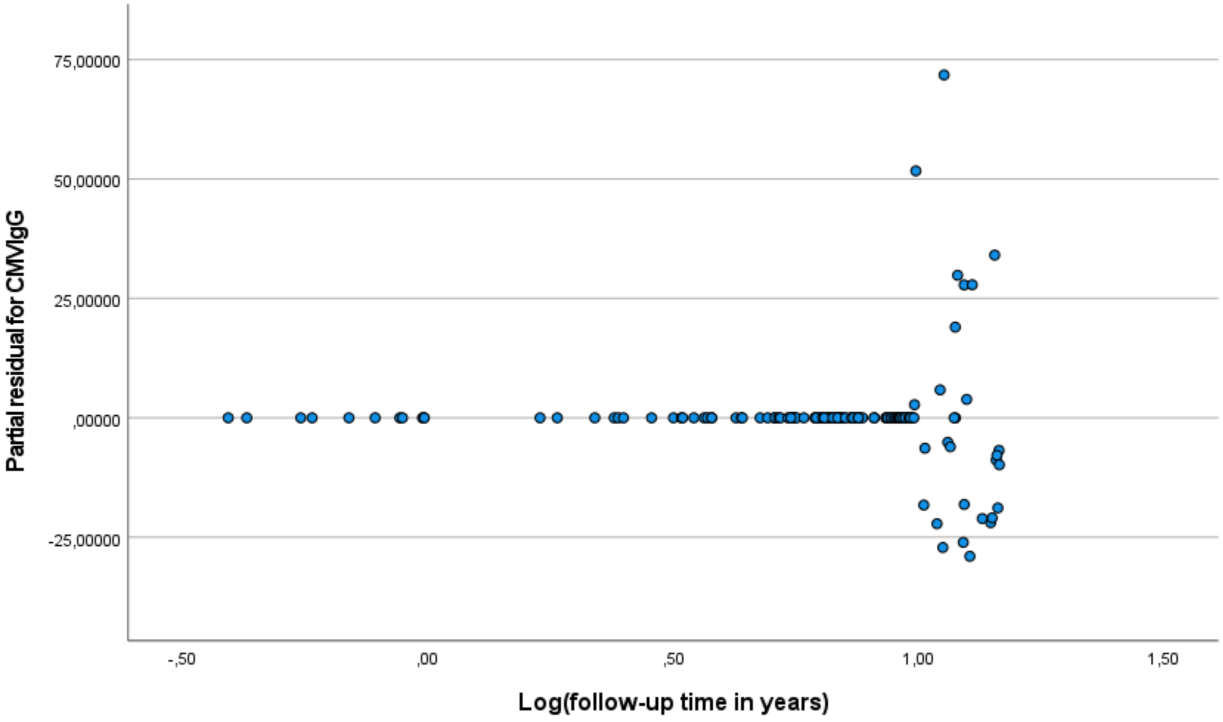

Supplementary Figure 4.2.1

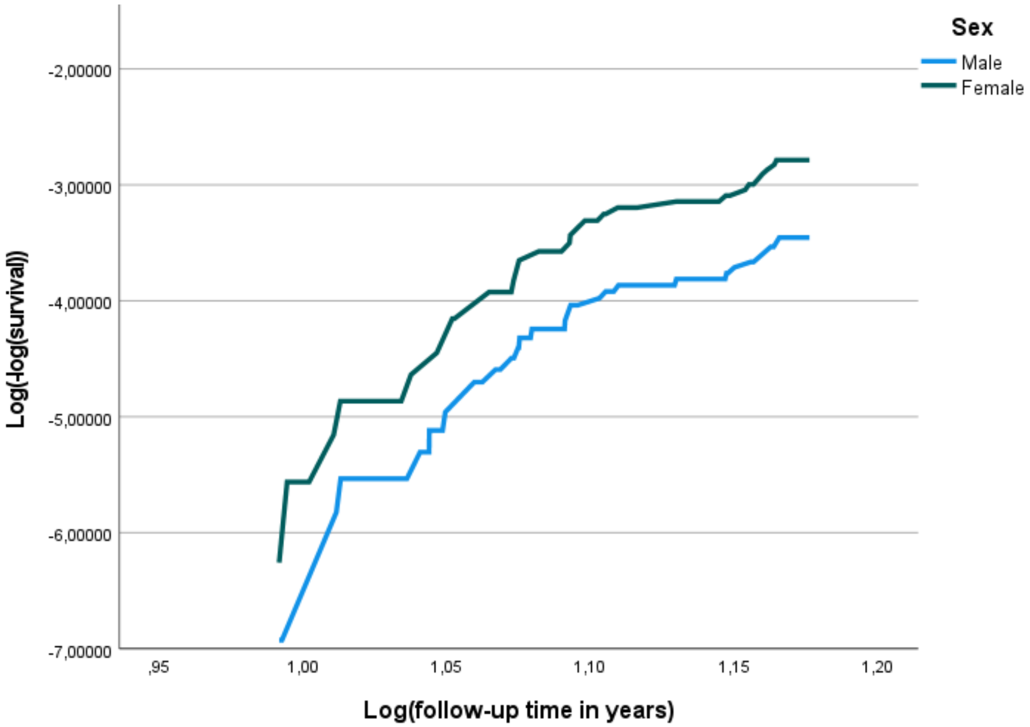

Supplementary Figure 4.2.2
